# Functional Identification of Close Proximity Amino Acid Side Chains within the Transmembrane-Spanning Helixes of the P2X2 Receptor

**DOI:** 10.1371/journal.pone.0070629

**Published:** 2013-08-06

**Authors:** Xin Liang, Huijuan Xu, Caiyue Li, Shikui Yin, Tingting Xu, Jinsong Liu, Zhiyuan Li

**Affiliations:** 1 Key Laboratory of Regenerative Biology, South China Institute for Stem Cell Biology and Regenerative Medicine, Guangzhou Institutes of Biomedicine and Health, University of Chinese Academy of Sciences, Guangzhou, Guangdong, China; 2 The School of Life Science, University of Science and Technology of China, Hefei, Anhui, China; 3 State Key Laboratory of Respiratory Disease, Guangzhou Institutes of Biomedicine and Health, University of Chinese Academy of Sciences, Guangzhou, Guangdong, China; University of Bern, Switzerland

## Abstract

The transition from the closed to open state greatly alters the intra- and inter-subunit interactions of the P2X receptor (P2XR). The interactions that occur in the transmembrane domain of the P2X2R remain unclear. We used substituted cysteine mutagenesis disulfide mapping to identify pairs of residues that are in close proximity within the transmembrane domain of rP2X2R and compared our results to the predicted positions of these amino acids obtained from a rat P2X2R homology model of the available open and closed zebrafish P2X4R structures. Alternations in channel function were measured as a change in the ATP-gated current before and after exposure to dithiothreitol. Thirty-six pairs of double mutants of rP2X2R expressed in HEK293 cells produced normal functioning channels. Thirty-five pairs of these mutants did not exhibit a functionally detectable disulfide bond. The double mutant H33C/S345C formed redox-dependent cross-links in the absence of ATP. Dithiothreitol ruptured the disulfide bond of H33C/S345C and induced a 2 to 3-fold increase in current. The EC_50_ for H33C/S345C before dithiothreitol treatment was ∼2-fold higher than that after dithiothreitol treatment. Dithiothreitol reduced the EC_50_ to wild-type levels. Furthermore, expression of trimeric concatamer receptors with Cys mutations at some but not all six positions showed that the more disulfide bond formation sites within the concatamer, the greater current potentiation after dithiothreitol incubation. Immunoblot analysis of H33C/S345C revealed one monomer band under nonreducing conditions strongly suggesting that disulfide bonds are formed within single subunits (intra-subunit) and not between two subunits (inter-subunit). Taken together, these data indicate that His33 and Ser345 are proximal to each other across an intra-subunit interface. The relative movement between the first transmembrane and the second transmembrane in the intra-subunit is likely important for transmitting the action of ATP binding to the opening of the channel.

## Introduction

P2X receptors are ATP-gated non-selective cation channels. In combination with widespread actions of ATP, P2X receptors, expressed on virtually every cell type [1], play essential roles in the body [2]. Thus, it is not surprising that P2X receptors mediate many physiological and pathological processes including synaptic transmission [3]-[7], pain signalling [8], the immune response [9]-[11], taste [12] and bone formation [13], which makes them attractive targets for drug discovery [14]-[18].

The crystal structure of the zebrafish P2X4.1 receptor (zfP2X4.1R) confirmed many mutagenesis-based predictions and for the first time provided a structural basis for directly studying the function of P2XRs at the molecular level. Substituted cysteine mutagenesis disulfide mapping has been used extensively to characterise intra- and inter-subunit contacts and has been valuable for studying the transmitting action of ATP binding to the opening of P2XR (Table 1). Disulfide mapping has identified several pairs of residues that sit close to each other across the inter-subunit interface; most of these pairs lie in the extracellular domain (Table 1). Hattori et al. [19] identified several intra- and inter-subunit interactions in the transmembrane domain (TMD) of the closed state of zfP2X4R. Several contacts exist between TM2 helices, including contacts between Leu340, Leu346, and Ala347, and the intra-subunit interactions are likely situated around a flexible hinge (located at Gly350) of TM2 [19]. When ATP activates the receptor, the two helices move away from the central axis by ∼3° to expand the ion permeating pore [19]. The interactions that stabilise the closed state of the pore are ruptured, and new contacts form to stabilise the opening state. Fifteen paired cysteine substitutions in the transmembrane domains were unable to form detectable disulfide bonds [20], [21]. The double mutant V48C/I328C is the only pair that has been demonstrated to form a disulfide bond in the TMD to date [21], but nevertheless suggests that movements between subunits are necessary for channel opening and presenting a useful method for studying the rearrangement of transmembrane helices from the closed to open states. Although the crystal structure of ATP-bound zfP2X4R provides a snapshot of the interactions in the TMD, a complete view of the interactions between the first transmembrane helix (TM1) and the second transmembrane helix (TM2) in both the closed and open states is an on-going goal for the field. One key question is whether these contacts between the transmembrane helices identified in the crystal structure of zfP2X4R exist in other subtypes of P2XR in different species and how they affect channel opening.

**Table 1:** Disulfide bond formation in P2X receptors.

Clone	Inter or intra	Effects	Subtype	Reference
K68C/F291C	Inter-subunit	ATP binding site in P2XRs	rP2X1R	[48], [49]
H120C/H213C	Inter-subunit	Inter-subunit Zn^2+^ binding site	rP2X2R	[50]
E167C/R290C	Inter-subunit	The distance between these two residues is less than 4.6 Å	rP2X2R	[51]
E59C/Q321C	Inter-subunit	Lateral fenestrations become larger when the channel opens	rP2X2R	[52]
E63C/R274C	Inter-subunit	ATP triggers relative movement of adjacent subunits head to tail	rP2X2R	[38]
V48C/I328C	Inter-subunit	Orientation of P2XR subunits; outward motion of each subunit	rP2X2R	[21]
K190C/N284C G60C/D320C I62C/L318C P196C/D320C P196C/K322C R197C/K322C F188C/N284C	Inter-subunit	ATP triggers relative movement of adjacent subunits	hP2X1R	[53]

The aim of this study was to identify other amino acids side chains lying in close functional proximity to one another and to compare their positions with those predicted by our P2X2R structural homology model, which is based on the available crystallographic data for the zfP2X4.1R [19]. Pairs of cysteines were introduced by mutagenesis into the TM1 and TM2 of rP2X2R, and interactions between the cysteines were probed by measuring the effect of the disulfide bond-reducing agent, dithiothreitol (DTT), on whole cell current amplitude. We demonstrate that one pair, His33 and Ser345, are proximal to each other across the intra-subunit interface. These results were further confirmed by Western blot, trimeric concatamers and energy coupling analysis.

## Materials and Methods

### Homology Model of the rP2X2 Receptor

Modelling of rP2X2R in the closed and open state was performed using the MODELLER module inside Discovery Studio 3.0 (Accelrys Inc.) with the crystal structures of zebrafish P2X4.1R (PDB ID 4DW0 for the closed state and 4DW1 for the open state) as the templates. The target and template share 49% sequence identity in the modelled region based on a BLAST alignment. The homology models of rP2X2R were refined and validated by VERIFY-3D (Discovery Studio 3.0, Accelrys Inc.) and MolProbity [22]; 99.3% of the residues in the closed model and 98.5% in the open model fall in the favoured regions of the Ramachandran diagram. The mutant models were built based on the closed form of the wild type model.

### Channel Constructs

Rat P2X2R clones were kindly provided by Dr. Terrance M. Egan (Saint Louis University). The FLAG-epitope (DYKDDDDK) was fused to the C terminus. The addition of the FLAG epitope has been shown to have no effect on the pharmacology [23] and function of P2XR [24], [25]. To remove the only native cysteine residues within the TMD (Fig. S1), we mutated Cys348 to threonine to create the rP2X2-T receptor (rP2X2R-T), which also closely functionally resembled the wild-type channel (Fig. S2). The FLAG-tagged rP2X2R-T construct was used as a template for the production of plasmids containing point mutations for specific amino acid residues using the KOD-Plus-Mutagenesis Kit (TOYOBO). Concatamers were constructed as previously described [26], [27] and confirmed by western blot. Primers for cloning and mutagenesis were synthesised by Invitrogen (Life Technologies). Each mutation was verified by an automated DNA sequencing service (Life Technologies). cDNAs were propagated in *Escherichia coli* DH5α, and plasmids were purified using the TaKaRa MiniBest Plasmid Purification Kit (TaKaRa).

### Transfection of HEK293 Cells

Human embryonic kidney cell line 293 (HEK293 cells) were used for the expression of wild type and mutant rP2X2R and routinely grown in Dulbecco’s modified Eagle’s medium (DMEM) with Glutamax (Invitrogen), 10% foetal bovine serum (HyClone), and antibiotics in a humidified 5% CO_2_ atmosphere. Trypsin-treated HEK293 cells were seeded in 6-well plates 1 d before transfection. Cells were prepared for transfection when confluence reached 70%-90%. The wild-type and mutant P2X2R expression vector were transiently coexpressed together with enhanced green fluorescent protein (EGFP) in HEK293 cells using Effectene Transfection Reagent (QIAGEN). For each transfection, 4 μl enhancer, 10 μl Effectene, 1 μg P2X2R cDNA and 1 μg EGFP cDNA were used according to the manufacturer’s instructions. The expression plasmid encoding EGFP was co-transfected to aid visual identification of transfected cells for electrophysiological recording experiments. Cells were used for whole-cell recording 24-48 h after transfection.

### Electrophysiological Recordings

Whole-cell currents were measured at room temperature from cells held at −60 mV using the perforated-patch, whole-cell, voltage-clamp technique [28], [29]. Whole-cell recordings were obtained with low resistance (2-4 MΩ) borosilicate glass electrodes that were pulled using a Flaming Brown Horizontal puller (P-97, Sutter Instruments) and were filled with 200 μg/ml amphotericin B dissolved in an intracellular solution with the following composition (in mM): 130 Cs-methanesulfonate, 24 CsCl, 1 MgCl_2_, 1 CaCl_2_, 10 HEPES. The composition of the extracellular solution was as follows (in mM): 154 NaCl, 1 MgCl_2_, 1 CaCl_2_, 10 glucose, and 10 HEPES, adjusted to pH 7.3 with NaOH. All solutions were maintained at pH 7.3–7.4 and 300–328 mOsm/L. All chemicals were purchased from Sigma. In all experiments, ATP and DTT were applied to single cells using RSC-200 Rapid Solution Changer (Biologic). Solution exchange occurred in 4 ms/tube. Solutions containing ATP were freshly prepared every 2 h. The timing of solution exchange was controlled by pClamp 10.0 software and standardised. Successive applications were separated by 2–5 min to minimise receptor desensitisation. Stabilisation of the pH of the drug is particularly important because P2X2R currents are augmented by acidification [30]. In whole-cell voltage clamp recordings, an Axonpatch 200B amplifier was controlled by pClamp 10.0 software via a Digidata 1440A interface board (Axon Instruments). Data were filtered at 2 kHz and digitised at 5 kHz.

### Preparation of the Membrane Fractions

Confluent cells were grown in T75 flasks. Forty-eight hours after transfection, we used a transmembrane protein extraction kit (Novagen) to isolate membrane fractions.

### Immunofluorescence

HEK293 cells were cultured on poly-L-lysine-coated coverslips. Cells were used at 24–48 h after transfection. Coverslips containing transfected cells were washed with phosphate-buffered saline (PBS) two times to remove DMEM medium. Next, the cells were fixed for 15 min at room temperature in 4% paraformaldehyde. The cells were then washed in PBS buffer three times (5 min each time) and permeabilised with 0.5% Triton X-100 in PBS for 15 min, after which they were washed in PBS three times (5 min each time). Subsequently, the cells were incubated in blocking buffer (1% BSA, PBS, pH 7.5) for 1 h to block nonspecific antibody binding. The cells were then incubated in blocking buffer containing primary antibody (anti-P2X2 antibody, 1:200, Abcam, USA) at 4 °C overnight or room temperature for 2 h. Next, the cells were washed with PBS five times (5 min each time), after which they were incubated in secondary antibody (Goat Anti-Mouse IgG-HRP, 1:2000, Abmart) for 30 min at room temperature. After washing with PBS, coverslips containing transfected cells were covered with antifade mounting medium (Beyotime, China) to prevent fluorescence fading. At last, the coverslips were sealed with nail polish. Fluorescence was visualised on a FV1000 Olympus epifluorescent microscope using a 40× oilimmersion objective. Images were acquired using a cool-snap HQ digital camera.

### Western Blot Analysis

The SDS-PAGE methods were as described previously [31]. Solubilised proteins were separated by SDS-PAGE (8% acrylamide gradient gel) and electrophoretically transferred to polyinylidene difluoride (PVDF) membranes. The PVDF membranes were blocked with PBST buffer (PBS containing 0.1% Tween 20) containing 5% nonfat milk at room temperature for 1 h. After washing with PBST, the PVDF membranes were incubated overnight with the primary antibody (mouse monoclonal anti-FLAG antibody, 1:2000, Abcam) at 4 °C. After washing with PBST five times, the membranes were incubated for 2 h with secondary antibody (horseradish peroxidase-conjugated goat anti-Mouse IgG, 1:5000, Abmart) at room temperature. Membranes were washed, and immunoreactive proteins were detected with Pierce ECL western blotting substrate (Thermo, USA) using Kodak BioMaxMS film (Kodak, Japan). The primary or secondary antibody was dissolved in antibody buffer (Beyotime, China). For each result, four independent experiments were repeated.

### Data Analysis

Concentration-response relationships for ATP were fitted by a Hill equation (SigmaPlot 10.0, SPSS Inc.) as follows:
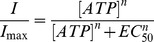
(1)where *I* and *I_max_* are the peak current of a given ATP concentration and the maximum current, respectively. [ATP] is the concentration of ATP. n_H_ is the Hill coefficient. EC_50_ is the concentration of ATP that gives a half-maximal response.

Free energy changes (ΔΔG) for the mutant (mut) were calculated according to
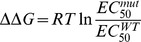
(2)where *R*  =  1.99 cal/mol/K, *T*  =  293K and EC_50_^mut^ and EC_50_^WT^ are the EC_50_ for the single or double mutant and rP2X2R-T, respectively.

The coupling energy of interaction between two mutants (ΔΔG_INT_) was calculated according to
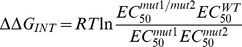
(3)where EC_50_^mut1/mut2^ is the EC_50_ of the double mutant. The experimental error of 2σ was calculated for two S.D. from the mean [32].

Data are the mean ± S.E.M. from at least three experiments. Significances were calculated using Student’s t test.

## Results

### Homology Modelling of rP2X2R and Initial Study

We generated homology models of the closed and open state of rP2X2R (residues 30-353) based on the crystal structures of the closed and open state of zfP2X4R (residues 32-361) using the MODELLER program [19]. Because this study is focused on the pore opening mechanism, we did not model the N and C termini, which were missing in the crystal structure of zfP2X4R in the open state. Here, we use rP2X2R numbering for each amino acid, unless otherwise stated.

We mutated native cysteine residues (Cys348) on TM2 to threonine (Fig. S1) and left the two native cysteine residues (Cys9 and Cys430) in the N and C termini unmutated, because our study focuses only on the pore segment. Previous experiments revealed that the FLAG-tagged rP2X2R-T is functional when expressed in HEK293 cells, and immunofluorescence indicated that rP2X2R-T was expressed at the plasma membrane at levels comparable to those of the rP2X2R-WT (Fig. S2A). The rise time and decay time of rP2X2R-T were highly similar to those of the rP2X2R-WT (Fig. S2B and C). In the presence of 30 μM ATP, rP2X2R-T desensitised slowly (Fig. S2D). The EC_50_ of rP2X2R-WT (EC_50_  =  4.1 ± 0.9 μM) and rP2X2R-T (EC_50_  =  3.7 ± 0.6 μM) were nearly identical (Fig. S2D and E). These results are consistent with previously published work showing that the triple mutant C9T/C348T/C340T (called P2X2R-3T) exhibited similar functional properties to rP2X2R-WT. These features of the rP2X2R-T make it an appropriate background for studying the pore opening mechanism with introduced cysteine residues.

### Effects of Introducing Pairs of Cysteines into the Transmembrane Domain

The proximity of the α-carbon atom (C_α_) between two residues within a protein must be less than 8.6 Å for disulfide bond formation [20], [28]. On the basis of the crystal structure of zfP2X4.1R and the homology model of rP2X2R in the closed state, we identified a series of residues that were predicted to be close enough to form disulfide bonds (< 9 Å between two residues C_α_). We studied the properties of 37 paired substitutions (including one positive control, V48C/I328C) (Table 2).

**Table 2:** Cysteine mutants in rP2X2 receptor.

Clone	*I*_basal_ (pA/pF)	*n*	Clone	*I*_basal_ (pA/pF)	*n*	Clone	*I*_basal_ (pA/pF)	*n*
rP2X2R-T	72.2 ± 10.9	20	Q37C with			F44C with		
G30C with			G342C	17.2 ± 2.5	19	I332C	69.9 ± 6.6	6
I328C*	N.T.		V343C	14.2 ± 1.4	5	L334C	40.6 ± 2.6	6
N333C*	N.T.		G344C	12.6 ± 1.8	8	A337C	28.8 ± 1.7	6
T336C*	N.T.		S345C	15.5 ± 3.5	8	I328C*	N.T.	
S345C	76.4 ± 14.0	7	I328C*	N.T.		T336C*	N.T.	
I351C*	N.T.		N333C*	N.T.		L338C*	N.T.	
L352C*	N.T.		T336C*	N.T.		N333C*	N.T.	
H33C with			L338C*	N.T.		Y47C with		
I341C	70.2 ± 11.9	8	I40C with			P329C	34.5 ± 2.3	6
G342C	53.9 ± 12.9	7	L334C	119.9 ± 12.2	5	N333C	123.8 ± 10.3	8
S345C	95.9 ± 12.3	30	L338C	135.4 ± 13.1	9	V48C with		
L347C	157.6 ± 21.2	6	S345C	130.3 ± 18.5	8	I328C	12.8 ± 1.8	40
C348	65.1 ±17.5	12	L41C with			P329C	22.8 ± 4.9	6
R34C with			L334C	50.3 ± 11.4	10	I332C	72.2 ± 10.2	6
G344C	5.8 ± 0.6	27	L338C	44.4 ± 9.5	6	N333C*	N.T.	
S345C	11.6 ± 2.4	8	Y43C with			T336C*	N.T.	
M35C with			I328C	4.3 ± 0.5	12	F49C with		
S345C	76.3 ± 11.2	10	I332C	13.1 ± 1.2	6	I332C	71.8 ± 8.9	5
V36C with			N333C	85.9 ± 7.4	13	V51C with		
S345C	81.7 ± 4.9	6	T336C	2.7 ± 0.7	5	I328C	57.6 ± 5.8	6
Q37C with			S340C	34.9 ± 8.8	45	S54C with		
S340C	107.6 ± 14. 8	6	G344C	5.6 ± 0.2	6	I328C	22.5 ± 4.0	5

The double mutations with asterisks are from previous studies [20], [21], which demonstrated that none of the double mutations formed disulfide bonds. N.T. means this double mutation was not tested. Data shown in the table are the mean ± S.E.M. from the cells studied, and the number of cells studied is given by *n*.

The whole cell currents produced by most double mutant receptors were between ∼900 to ∼2500 pA, comparable to the values for the rP2X2R-T. For double mutants containing Q37C, the currents were < 300 pA (150-300 pA, *n* > 6). For double mutants containing R34C, the currents were < 200 pA (60-200 pA, *n* > 5). For double mutants containing Y43C, the currents were < 100 pA (*n* > 6). The current amplitudes of the double mutants containing Q37C, R34C, or Y43C were less than 25% of that recorded for the wild type and were consistent with the smaller currents of the single mutants Q37C, R34C, and Y43C [21], [33]. The current densities of Q37C/S340C (107.6 ± 14.8 pA/pF, *n*  =  6) and Y43C/N333C (85.9 ± 7.4 pA/pF, *n*  =  13) were comparable to that of rP2X2R-T (72.2 ± 10.9 pA/pF, *n*  =  20), but the mechanism is unknown. For H33C/G342C, the whole cell current density was 53.9 ± 12.9 pA/pF (n  =  7), and the desensitisation was faster than that of the rP2X2R-T.

### Disulfide Bond Formation between H33C and S345C

**Figure 1:**
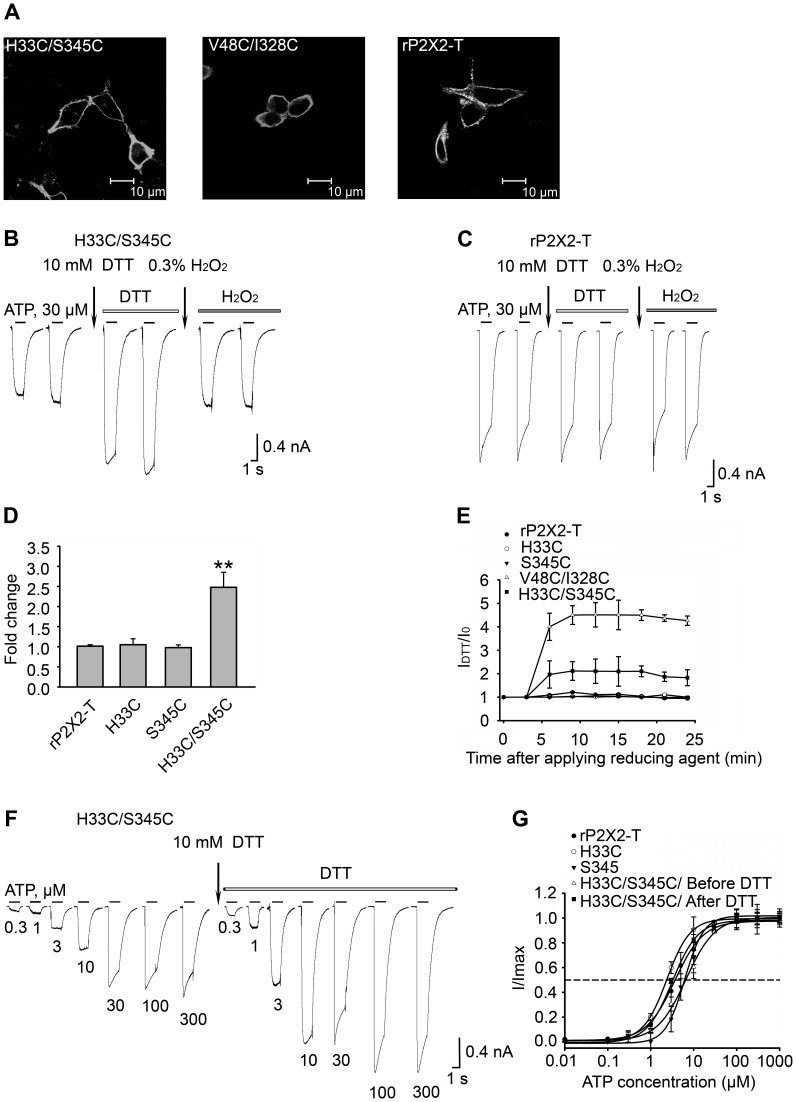
Disulfide bond formation between H33C and S345C alters channel opening. (A) Subcellular distribution of H33C/S345C (left panel), V48C/I328C (middle panel) and rP2X2-T (right panel) 24 h after transfection in the HEK293 cell line. Scale bar is 10 µm. (B) Effect of DTT and H_2_O_2_ on the H33C/S345C double mutant. After two stable responses were evoked by 30 μM ATP (black bar), the cells were incubated in 10 mM DTT for 5 min (first arrow) and were then evoked by 30 μM ATP plus 10 mM DTT (white bar). After stable currents were obtained, cells were incubated with 0.3% H_2_O_2_ (second arrow) for 3 min to reverse the effects of DTT, after which the cells were evoked by 30 μM ATP plus 0.3% H_2_O_2_ (grey bar). (C) The same protocol was applied to the rP2X2R-T, and had no effect on the responses evoked by 30 μM ATP plus 10 mM DTT. (D) Summary of relative current change in H33C/S345C and rP2X2R-T after DTT application. ** (*P*< 0.01), the values are significantly different from those obtained for H33C, S345C and rP2X2R-T. (E) Time course of the potentiation of ATP-evoked currents in V48C/I328C (△) and H33C/S345C (▪) double mutants by DTT. rP2X2R-T (•), H33C (○) and S345C (▾) single mutants were not affected by treatment with DTT. (F) Different concentrations of ATP (black bar) evoke currents in H33C/S345C. Each concentration of ATP (indicated below recordings) was applied twice for 2 s with similar results. 30 μM ATP was applied before each test concentration to evaluate rundown. The cell was superfused with 10 mM DTT (indicated by an arrow) for 5 min, and ATP plus DTT (white bar) were then co-applied for 2 s to evoke an inward current. DTT induced changes upon comparison with the control condition. (G) Concentration-response curves generated from the same experiment in (F) for rP2X2R-T (•), H33C (○), S345C (▾), H33C/S345C before (△) and after DTT application (▪). The EC_50_ curves of single mutant and rP2X2-T after DTT treatment are not shown for the sake of clarity, because there were no significant changes. The dotted line indicates that the value of I/I_max_ is equal to 0.5. For (D) and (E), all currents were normalised to those measured prior to application of DTT (*n*  =  3-10 cells for each case). For (B), (C) and (F), the gaps indicate 3-min time intervals between each ATP application.

We next tested the effect of DTT (10 mM, 3-25 min) on the ATP-gated current amplitudes of each of the 36 double cysteine mutants (Table 2), but only one mutant, H33C/S345C, displayed a significant change in current amplitude in response to exposure to this reducing agent. Previous studies have presented evidence that Ser345 (Asn353 in zfP2X4R) is located at the narrowest part between the TM1 and TM2 domains [19], [34], [35]. Therefore, we determined if Ser345 was sufficiently close to form disulfide bonds with residues on TM1 that would alter the properties of the channel and whether there is a difference between a disulfide bond formed in the inner TMDs and the V48C/I328C bond situated at the outer edge of the transmembrane domains. We expressed some of the double mutants near this narrowest part (Table 2 and Fig. S3) and observed that H33C and S345C were sufficiently close to form a disulfide bond. The currents elicited by 30 μM ATP in the H33C/S345C receptor were much larger (95.9 ± 12.3 pA/pF, *n*  =  30) than those in rP2X2R-T. Immunofluorescence experiments showed that H33C/S345C receptors are normally targeted to the cell membrane (Fig. 1A). Incubation of cells expressing H33C/S345C receptors in DTT (10 mM) for 5 min increased the ATP-gated current amplitude elicited by ATP by 2.48 ± 0.4-fold (Fig. 1B), whereas the same treatment had no significant effect on rP2X2R-T (Fig. 1C) and the single mutants, H33C and S345C (Fig. 1D). This finding suggests that DTT serves as a reducing agent to break the disulfide bond formed between H33C and S345C in the double cysteine mutant. After 20 min incubation in DTT, the amplitude of the current was progressively reduced due to receptor desensitisation (Fig. 1E). After DTT was removed, the increase in responsiveness to ATP lasted over 2 h, presumably because the cell surface was not sufficiently oxidizing to reform the disulfide bonds once they were broken. However, after 3 min incubations in 0.3% hydrogen peroxide (H_2_O_2_) the current amplitude was restored to its initial state before DTT application (Fig. 1B), suggesting successful reformation of the disulfide bonds. Furthermore, the ATP EC_50_ before DTT treatment (EC_50 before DTT_  =  7.3 ± 1.1 μM, *n*  =  10) was ∼2-fold higher than that after DTT treatment (EC_50 after DTT_  =  3.19 ± 0.3 μM, *n*  =  10) (Fig. 1F and G). Interestingly, the EC_50_ value of H33C/S345C after DTT treatment was indistinguishable from that of rP2X2R-T (Table 3). However, the EC_50_ value after H_2_O_2_ treatment (EC_50 after H2O2_  =  6.4 ± 0.5 μM, *n*  =  5) returned to the initial EC_50_ level before DTT application (Table 3). As with DTT, H_2_O_2_ had no effect on rP2X2R-T or on the single cysteine mutants, H33C and S345C (Fig. 1C and D). The ratio of the EC_50_ before DTT application to the EC_50_ after DTT application for H33C/S345C (2.4 ± 0.35) was significantly different (*P* < 0.05) from those observed for H33C (1.0 ± 0.04), S345C (1.1 ± 0.05) and rP2X2-T (0.9 ± 0.03). These results suggest that H33C and S345C were sufficiently close to form a disulfide bond, and that the presence of this bond impairs normal P2XR channel opening in response to agonists.

**Table 3:** Functional properties of cysteine mutant receptors.

Mutants	EC_50_ (*μM*)	*n*_H_	I_max_ (*pA/picofarad*)	*n*	ΔΔG (*Kcal.mol^-1^*)	ΔΔG_INT_ (*Kcal.mol^-1^*)
rP2X2R-WT	4.1 ± 0.9	0.7 ± 0.1	49.1 ± 10.6	20	0	
rP2X2R-T	3.7 ± 0.6	1.3 ± 0.3	51.7 ± 9.3	30	0	
V48C	5.8 ± 0.5	1.3 ± 0.1	66.8 ± 9.1	5	0.24 ± 0.03	
I328C	3.9 ± 0.6	1.5 ± 0.3	73.1 ± 11.4	5	0.09 ± 0.02	
H33C	2.3 ± 0.5	1.3 ± 0.3	35.8 ± 8.3	5	-0.29 ± 0.13	
S345C	6.3 ± 0.9	1.2 ± 0.1	40.5 ± 7.6	5	0.30 ± 0.08	
V48A	3.2 ± 0.6	1.6 ± 0.1	25.4 ± 2.1	7	-0.1 ± 0.11	
I328A	0.4 ± 0.1	1.7 ± 0.2	31.7 ± 6.1	6	-1.31 ± 0.15	
H33A	4.2 ± 0.6	1.6 ± 0.3	41.7 ± 8.3	6	0.08 ± 0.07	
S345A	12.1 ± 0.7	1.3 ± 0.2	27.5 ± 5.8	7	0.69 ± 0.03	
F44C	0.81 ± 0.1	0.9 ± 0.3	39.2 ± 2.1	4	-0.89 ± 0.07	
A337C	6.2 ± 0.5	1.2 ± 0.4	30.1 ± 4.1	4	0.30 ± 0.05	
V48C/I328C	17.8 ± 2.0	0.9 ± 0.2	12.8 ± 1.8	28	0.91 ± 0.07	0.59 ± 0.03
H33C/S345C	7.3 ± 1.1	1.3 ± 0.2	95.9 ± 12.3	10	0.36 ± 0.10	0.33 ± 0.10
V48A/I328A	5.4 ± 0.4	1.3 ± 0.3	42.0 ± 6.5	5	0.22 ± 0.04	1.60 ± 0.04
H33A/S345A	35.7 ± 0.5	1.4 ± 0.1	33.1 ± 4.1	7	1.32 ± 0.01	0.66 ± 0.01
F44C/A337C	1.5 ± 0.5	1.5 ± 0.2	41.4 ± 1.5	4	-0.52 ± 0.19	0.10 ± 0.18
rP2X2R-T after DTT	3.9 ± 0.5	0.7 ± 0.2	67.3 ± 11.2	10	0	
V48C after DTT	5.5 ± 0.5	1.3 ± 0.1	67.1 ± 8.7	5	0.23 ± 0.05	
I328C after DTT	4.0 ± 0.6	1.6 ± 0.3	70.2 ± 14.3	5	0.1 ± 0.03	
H33C after DTT	3.1 ± 0.3	1.3 ± 0.3	35.8 ± 8.3	5	-0.11 ± 0.06	
S345C after DTT	6.5 ± 0.7	1.2 ± 0.1	40.5 ± 7.6	5	0.32 ± 0.13	
V48C/I328C after DTT	3.6 ± 0.4	1.1 ± 0.2	21.1 ± 4.6	15	0.27 ± 0.19	-0.31 ± 0.07
V48C/I328C after H_2_O_2_	17.9 ± 1.9	0.7 ± 0.1	11.9 ± 1.7	6	0.92 ± 0.06	0.59 ± 0.03
H33C/S345C after DTT	3.19 ± 0.3	1.4 ± 0.2	97.2 ± 11.9	10	-0.09 ± 0.05	-0.12 ± 0.05
H33C/S345Cafter H_2_O_2_	6.4 ± 0.5	1.4 ± 0.3	63.8 ± 7.8	5	0.32 ± 0.05	0.28 ± 0.05

The data represent the mean ± S.E.M. of the numbers of cells studied (n).

**Figure 2:**
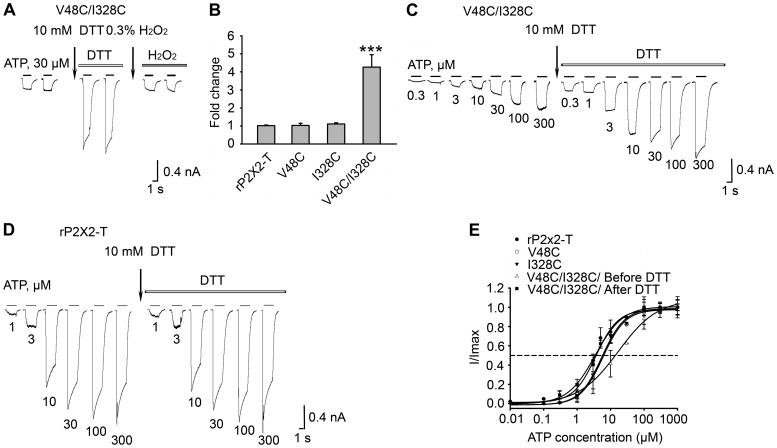
Disulfide bond formation between V48C and I328C alters channel opening. (A) Effect of DTT and H_2_O_2_ on V48C/I328C double mutant. The same protocol of Figure 1B was applied to this double mutant. Application of DTT caused a ∼4-fold increase in receptor current. Application of 0.3% H_2_O_2_ reversed the effect of DTT. (B) Summary of relative current change in V48C/I328C and rP2X2R-T after DTT application. *** (*P*< 0.001), values were significantly different from those obtained for V48C, I328C and rP2X2R-T. For (B), all currents were normalised to those measured prior to application of DTT (*n*  =  3-10 cells for each case). Figure (C) and (D) show that different concentrations of ATP evoke currents in V48C/I328C and rP2X2R-T, respectively. Both were applied the same protocol as described in Figure 1F. (E) Concentration-response curves generated from same experiment in (C) and (D) for rP2X2R-T (•), V48C (○), I328C (▾) and V48C/I328C before (△) and after DTT application (▪). The EC_50_ curves of single mutant and rP2X2-T after DTT treatment are not shown for the sake of clarity, because there were no significant changes. The dotted line indicates that the value of I/I_max_ is equal to 0.5. For (C) and (D), the gaps indicate 3-min time intervals between each ATP application.

For comparison, we applied the same protocol to cells expressing V48C/I328C, which has already been reported to form inter-subunit disulphide bonds [36]. We occasionally observed currents that were larger (> 900 pA) or smaller (< 50 pA) than the average level, which may be related to intrinsic cellular conditions that affected the expression level of the receptor. DTT greatly increased the amplitude of the current evoked by ATP by 4.26 ± 0.7-fold over 25 min (Fig. 2A and B) and progressively reduced because of the desensitization (Fig. 1E). The current amplitude elicited by different ATP concentrations was much smaller (Fig. 2C) (30 μM ATP, 12.8 ± 1.8 pA/ pF, n  =  40) than that of rP2X2R-T (Fig. 2D and Table 2), even though the double mutant was normally targeted to the cell membrane (Fig. 1A). More surprising, the EC_50_ before DTT (17.8 ± 2.0 μM, *n*  =  28) was ∼5-fold greater than that after DTT (3.6 ± 0.4 μM, *n*  =  15) (Fig. 2C and 2E), and treatment with H_2_O_2_ caused the EC_50_ value to return to its original level (EC_50 after H2O2_  =  17.9 ± 1.9 μM, *n*  =  6) (Table 3). The ratio of the EC_50_ before DTT application to the EC_50_ after DTT application for V48C/I328C (4.8 ± 0.5) was significantly different (*P* < 0.01) from those observed for V48C (1.0 ± 0.03), I328C (1.0 ± 0.06) and rP2X2-T (0.9 ± 0.03). These results suggest that disulfide bond formation hindered subunit movement and resulted in reduced P2XR opening.

### Intra-subunit Disulfide Bond Formed between H33C and S345C

**Figure 3:**
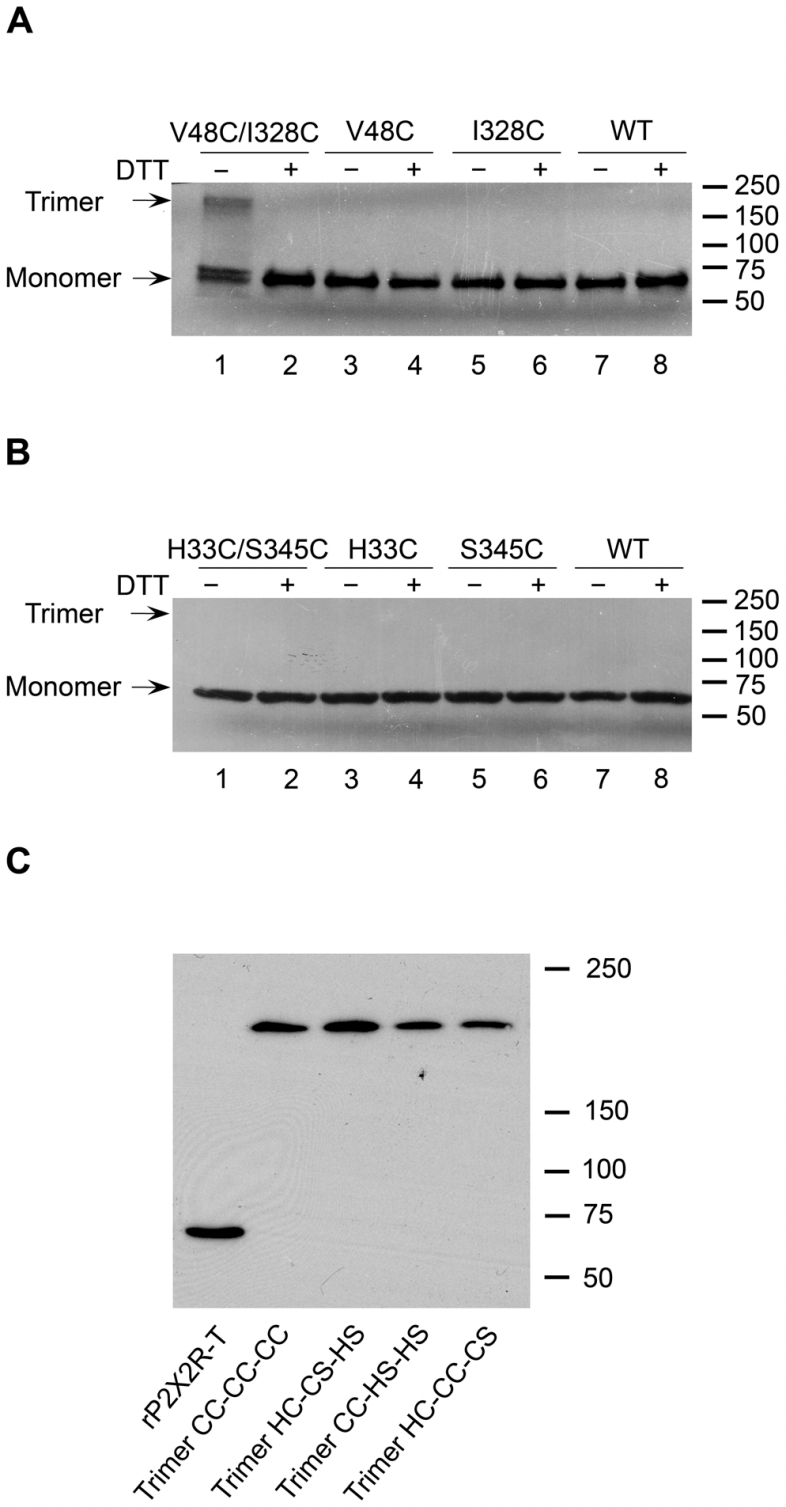
Western blot analysis. (A) Inter-subunit disulfide bond formation between V48C and I328C in the rP2X2R. Double mutant V48C/I328C, single mutants V48C and I328C and wild-type rP2X2R were transiently expressed in HEK293 cells. Protein samples were extracted from the membrane. (B) Analysis of specific trimer formation in double mutant H33C/S345C, single mutants H33C and S345C and wild-type rP2X2R. In (A) and (B), all the single mutants and the wild type protein served as negative controls to estimate the background of nonspecific disulfide bond formation. Arrows indicate monomers and trimers. Above lanes 2, 4, 6, and 8 in (A) and (B), “+” means protein samples were loaded with DTT to denature the disulfide bond. Above lanes 1, 3, 5, 7 in (A) and (B), “–” means protein samples were loaded without DTT. Proteins were separated on SDS-PAGE gels (8%) and detected by Western blotting via a FLAG-tag antibody. Protein molecular weight markers (kDa) are indicated on the right. These results were observed in at least four independent experiments for each receptor. (C) Western blot analysis of the concatamerised trimers. The rP2X2R-T monomer, trimers CC-CC-CC, CC-HS-HS, HC-CS-HS, and HC-CC-CS were transiently expressed in HEK293 cells. H and S mean His33 and Ser345, respectively. C means cysteine substitution. In the monomer, each subunit has one N terminus and one C terminus. The concatameric trimer constructs have only one N terminus and one C terminus. Subunit organizations of concatameric trimer constructs are presented in Figure 4A. Protein samples were extracted from the membrane, separated by SDS-PAGE gels (8%) under reducing conditions, and detected by Western blotting with rP2X2 antibody. The positions of molecular mass standards (kDa) are shown on the right. The trimers revealed a single band indicating the same size (∼186 kDa) and remained intact. These results were observed in at least four independent experiments for each receptor.

Inter- and intra-subunit disulfide bond formation could have different effects on P2XR channel activity. To determine if the disulfide bond formed between H33C and S345C occurs between two neighbouring subunits (inter-subunit), as is the case with V48C/I328C, we extracted receptor protein from the membrane after expressing wild-type and mutant rP2X2R in HEK293 cells. The rP2X2R-WT subunits as well as subunits containing V48C or I328C substitutions alone primarily migrated on SDS-PAGE to the position expected for the monomeric subunit (∼62 kDa; monomer arrowhead in Fig. 3A) under reducing (addition of 20 mM DTT to the protein sample) or nonreducing conditions. In the case of V48C/I328C, due to its inter-subunit disulfide bond formation, the trimer (∼186 kDa; trimer arrowhead in Fig. 3A) was observed as expected based on previous work, which was reduced to the monomer under reducing conditions. However, the subunits containing H33C or S345C substitutions alone as well as the double mutant H33C/S345C predominantly migrated on SDS-PAGE to the monomer position (Fig. 3B); in this case, no dimer or trimer was formed. This finding suggests that the disulfide bond in H33C/S345C is formed within a single subunit (intra-subunit), which supports the predictions of our P2X2R homology model and is consistent with the crystal structure of zfP2X4.1R and previous studies [19], [34], [35].

**Figure 4:**
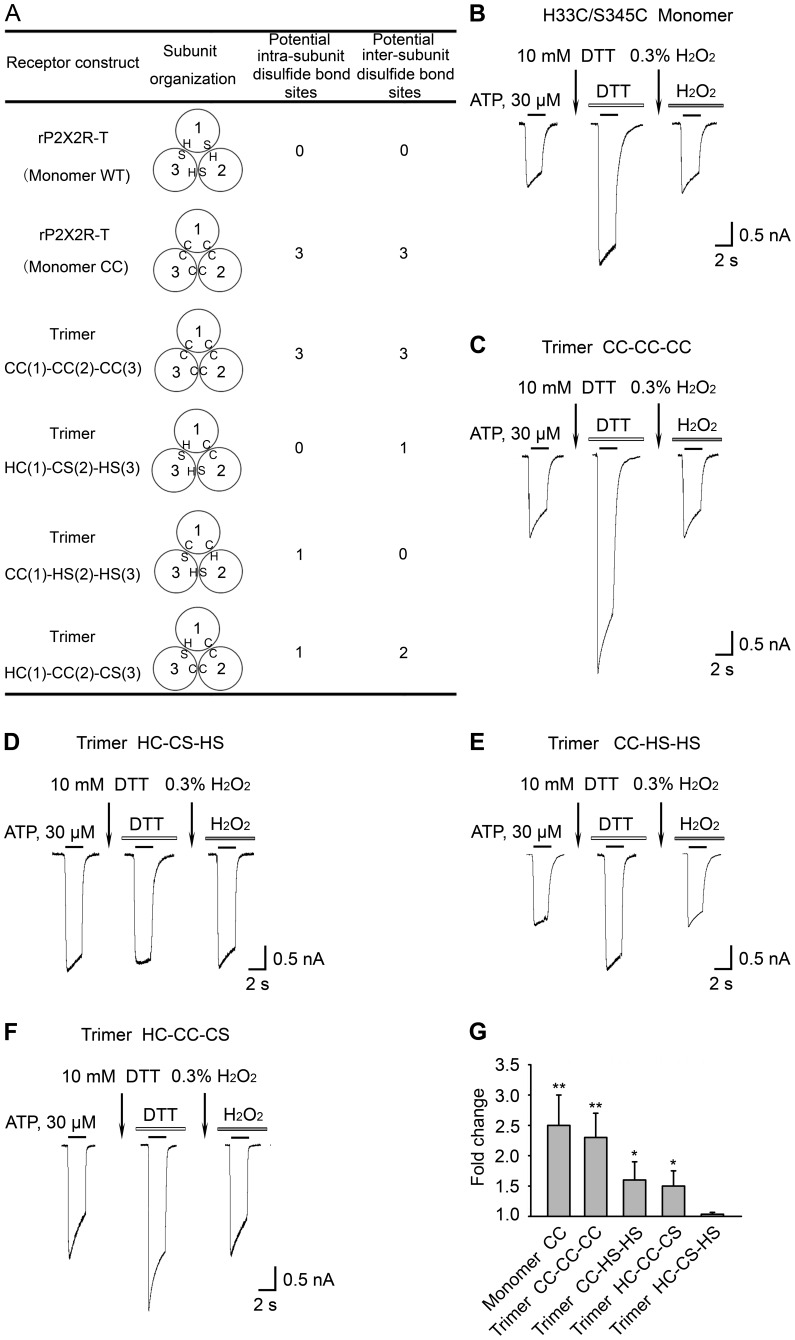
Concatameric constructs suggest an intra-subunit interaction. (A) Predicted number of intra-subunit and inter-subunit disulfide bond sites in the receptor construct. In each diagram, H and S mean His33 and Ser345, respectively. C means cysteine substitution. A circle indicates one subunit. Three subunits make up a receptor and are numbered 1, 2 and 3. In the monomer, each subunit has one N terminus and one C terminus. The concatameric constructs have only one N terminus and one C terminus. Figures (B), (C), (D), (E) and (F) present the effects of DTT and H_2_O_2_ on the H33C/S345C monomer, trimer CC-CC-CC, trimer HC-CS-HS, trimer CC-HS-HS, and trimer HC-CC-CS, respectively. After stable responses were evoked by 30 μM ATP (black bar), the cells were incubated in 10 mM DTT for 5 min (first arrow) and were then evoked by 30 μM ATP plus 10 mM DTT (white bar). After stable currents were obtained, cells were incubated with 0.3% H_2_O_2_ (second arrow) for 3 min to inverse the effects of DTT, after which the cells were evoked by 30 μM ATP plus 0.3% H_2_O_2_ (grey bar). The gaps indicate 3-min time intervals between ATP applications. The same protocol was applied to the H33C/S345C monomer and four different concatameric constructs. For (B), (C), (D), (E), and (F), all currents were measured at least twice to obtain stability. (G) Summary of relative current changes in (B), (C), (D), (E), and (F) after DTT application. All currents were normalised to those measured prior to application of DTT (*n*  =  3-10 cells for each case). For (G), * (*P*< 0.05), values are significantly different from that observed for trimer HC-CS-HS. ** (*P*< 0.01), values are significantly different from that observed for trimer HC-CS-HS.

We next made a series of concatameric receptors by splicing three coding units together. The trimers were constructed from rP2X2R monomers. To determine whether rP2X2R concatamers are expressed as full-length trimers, proteins from HEK293 cells expressing rP2X2R-T or trimers (CC-CC-CC, CC-HS-HS, HC-CS-HS, HC-CC-CS) were subjected to SDS-PAGE and immunoblot analysis (Fig. 3C). H and S indicate as His33 and Ser345, respectively. C indicates as cysteine substitution at positions 345 or 33. In the monomer, each subunit has one N terminus and one C terminus. The concatameric constructs have only one N terminus and one C terminus (Fig. 4A). A single protein band was present at ∼186 kDa for all four concatameric receptors, indicating that they were processed into full-length trimers (Fig. 3C).

All of our trimeric constructs were functional (Fig. 4). To determine whether an intra-subunit disulfide bond was present, we used the same protocol used in Fig. 1B. The increase in current amplitude observed after DTT incubation for the concatamer with all six cysteine mutations (trimer CC-CC-CC) was not significantly different from that observed for the receptor made up of three H33C/S345C monomers assembled independently (Fig. 4B and C). For CC-CC-CC, the current amplitude increased ∼2.6 fold in response to DTT, while, for the H33C/S345C monomer, the amplitude increased ∼2.2 fold. Consistent with the hypothesis that the disulfide bond of H33C/S345C is formed within single subunit (intra-subunit), the concatamer with H33C in subunit 2 and S345C in subunit 1 (trimer HC-CS-HS) (Fig. 4D) demonstrated no current amplitude potentiation after DTT incubation. In contrast, the concatamer with two cysteines in a single subunit (trimer CC-HS-HS) (Fig. 4E) showed potentiation after DTT incubation (the current amplitude increased ∼1.6 fold) that was similar to that observed for the trimer HC-CC-CS (for which the current amplitude increased, ∼1.6 fold) (Fig. 4F and G). For the trimers CC-CC-CC, CC-HS-HS, and HC-CC-CS, after 3 min incubations in 0.3% hydrogen peroxide (H_2_O_2_), the current amplitudes were restored to their initial states before DTT application. Because these three trimers are predicted to have 3, 1, and 1 intra-subunit disulfide bond formation sites respectively (Fig. 4A), it was of interest to compare current amplitude potentiations after DTT incubation in these constructs (Fig. 4G). The monomer CC and trimer CC-CC-CC have similar changes in current amplitudes, which are significantly different from the results obtained for the trimers CC-HS-HS, HC-CC-CS, and HC-CS-HS. However, the trimer CC-HS-HS and HC-CC-CS have similar changes in current amplitudes (Fig. 4G). Because they are each predicted to have one intra-subunit disulfide bond (Fig. 4A), the trimer CC-HS-HS and HC-CC-CS both demonstrated weak current increases. The concatameric trimer experiments suggest that the disulfide bond in H33C/S345C is predominantly formed within single subunits (intra-subunit) rather than between two subunits (inter-subunit). This, and the observation that the double mutant H33C/S345C was functional but exhibited a weaker current increase after DTT application when compared to V48C/I328C also supports our P2X2R homology model’s prediction that the proximity of His33 and Ser345 does not change so much during channel gating as seems to be the case for the inter-subunit proximity of Val48 and Ile328.

### Non-additive Effects of Double Mutants of rP2X2R

Double mutant cycle analysis is a commonly used approach that enables us to quantify the energetics of the interactions between residues on the basis of the free energy changes (ΔΔG) associated with a perturbation without being biased by structural information about the interface [32], [37]. It has been used to investigate ligand-gated ion channels [38], [39]. The conventional procedure for experimental analysis is site-directed mutagenesis. If the two mutated residues are energetically coupled (co-operative), then the change in free energy of the double mutant is different from the sum of the free energies of the two single mutants, indicating a specific interaction between them. ΔΔG_INT_ is a coupling energy that measures the co-operative interaction of the two mutated residues.

**Figure 5:**
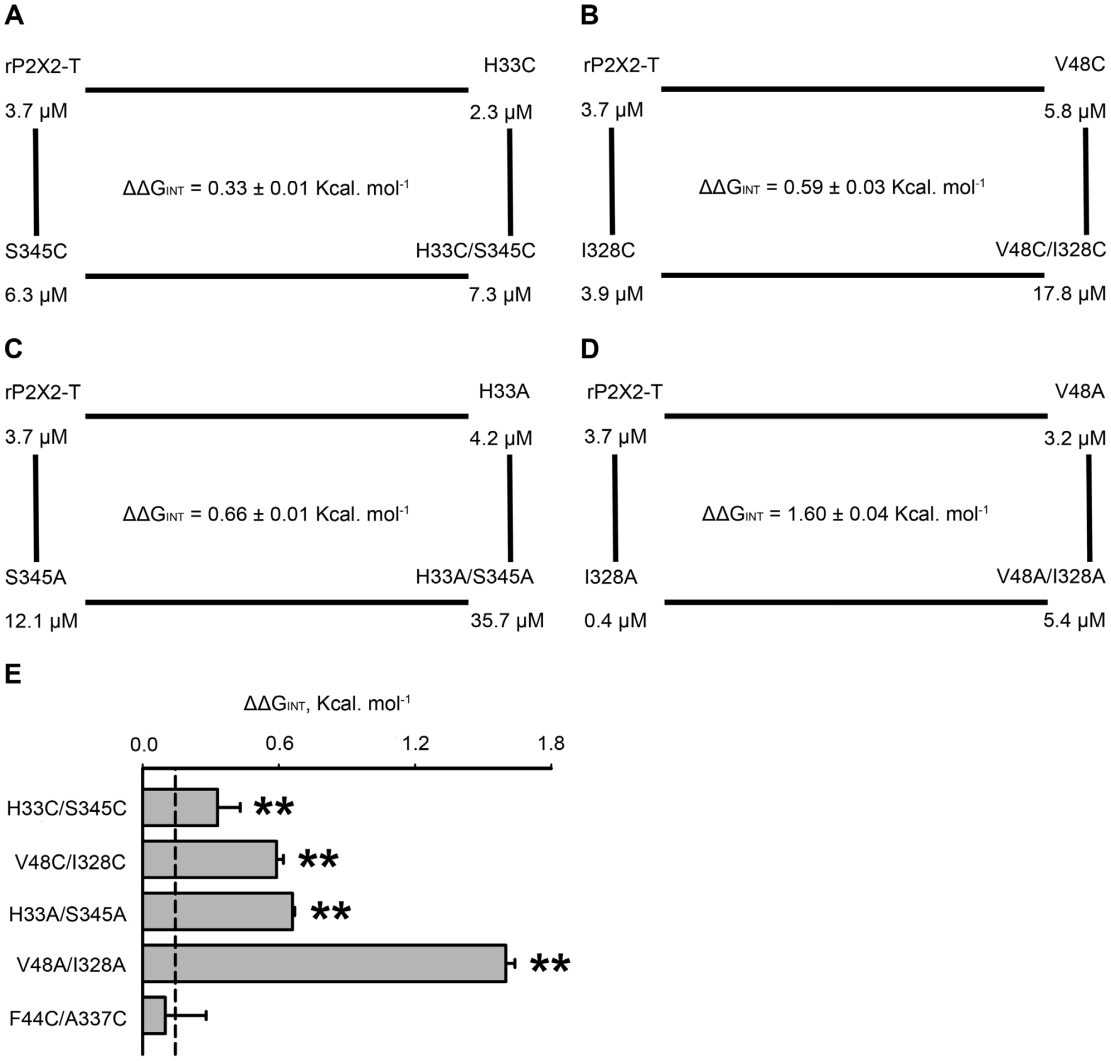
Double mutant cycle analysis for His33 and Ser345. (A) Mutant cycle analysis shows free energy changes between H33C and S345C. (B) Mutant cycle analysis shows free energy changes between V48C and I328C. (C) Mutant cycle analysis shows free energy changes between H33A and S345A. (D) Mutant cycle analysis shows free energy changes between V48A and I328A. (E) Histogram showing the calculated coupling energy (ΔΔG_INT_) for the indicated pairs, H33C/S345C, V48C/I328C, H33A/S345A, V48A/I328A and F44C/A337C. The dashed line indicates the experimental error (2σ), which corresponds to ± 0.14 kcal/mol. ** (*P*< 0.01), values are significantly different from those observed for negative control F44C/A337C.

ΔΔG_INT_ is small but significant for the pair H33C/S345C. The free energy is not the sum of the free energies of H33C and S345C, suggesting a strong interaction between His33 and Ser345 (Fig. 5A and Table 3). To further confirm this strong interaction in H33C/S345C, we used V48C/I328C and F44C/A337C as positive and negative controls, respectively. A significantly higher value of ΔΔG_INT_ was calculated for V48C/I328C (ΔΔG  =  0.91 ± 0.07 Kcal.mol^-1^; ΔΔG_INT_  =  0.59 ± 0.03 Kcal.mol^-1^) (Fig. 5B and Table 3).

Alanine mutations are reliable for the analysis of a double mutant cycle because substitution with alanine abolishes interactions without the formation of new interactions [40]. The results of non-alanine and alanine double-mutant cycle analysis could corroborate each other and further confirm our results. We therefore introduced alanine mutations into the rP2X2R constructs. As expected for interacting residues, the ΔΔG values for H33A/S345A (ΔΔG  = 1.32 ± 0.01 Kcal.mol^-1^; ΔΔG_INT_  =  0.66 ± 0.01 Kcal.mol^-1^) (Fig. 5C and Table 3) and V48A/I328A (ΔΔG  =  0.22 ± 0.04 Kcal.mol^-1^; ΔΔG_INT_  =  1.60 ± 0.04 Kcal.mol^-1^) (Fig. 5D and Table 3) were not the additive sums of the ΔΔG calculated from the respective single mutants. By contrast, the ΔΔG_INT_ value for F44C/A337C, as expected, was not significant and was close to the experimental error (Fig. 5E and Table 3). The ΔΔG_INT_ values for H33C/S345C, H33A/S345A, V48C/I328C, and V48A/I328A were significantly different from F44C/A337C (Fig. 5E). These data suggest that the side chains at positions His33 and Ser345 structurally interact at the intra-subunit interface between TM1 and TM2.

### Coordinating Residues at Ser345 for Metal Bridges Formation

**Figure 6:**
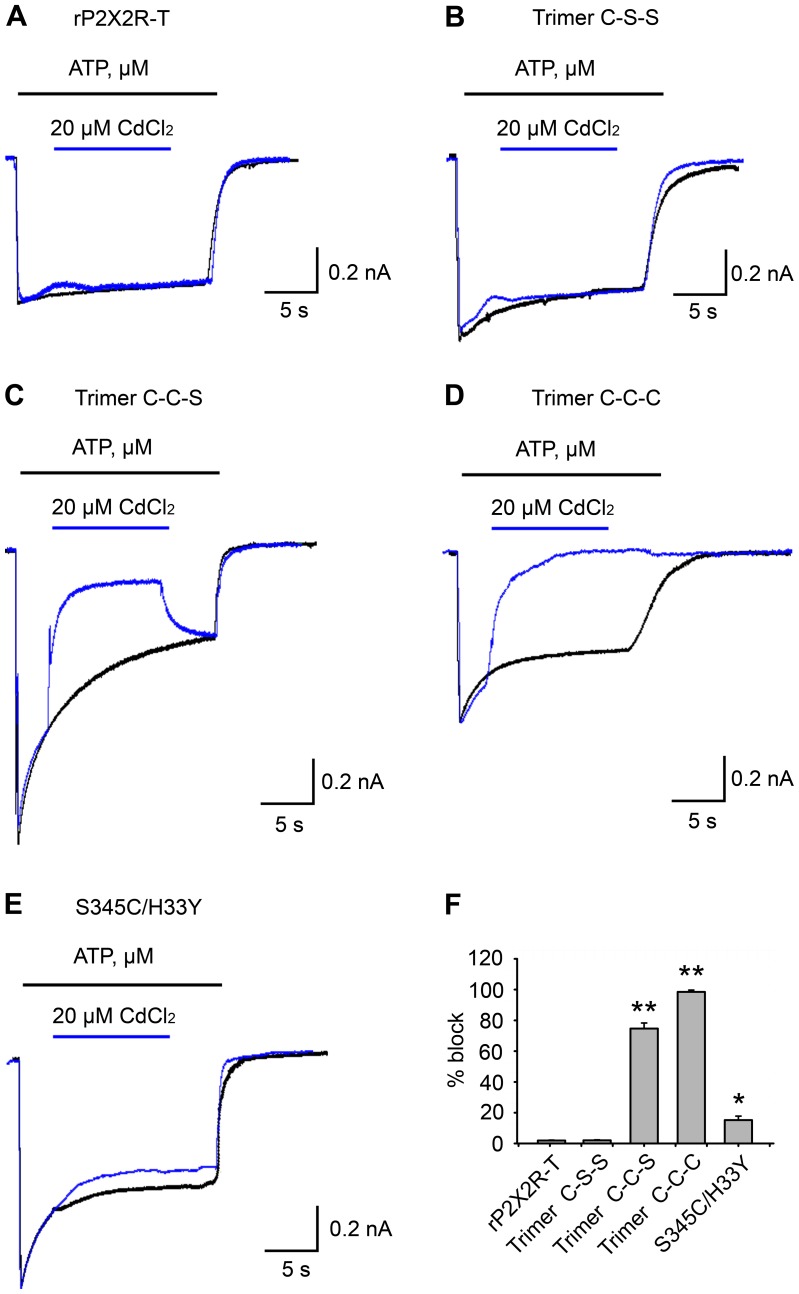
Coordinating residues at Ser345 for metal bridge formation. (A) Superimposed scaled current traces show that rP2X2R-T currents are not inhibited by applying 20 μM CdCl_2_. The control current trace (black) is evoked only by 30 μM ATP. For the test current trace (blue), 30 μM ATP was applied for 5 s, after which the solution was switched to one containing 30 μM ATP plus 20 μM Cd^2+^ for 10-20 s. Following this, we returned the cell to a solution containing only 30 μM ATP for 5 s. The same protocol was applied to the other constructs in (B), (C), (D), and (E). In (B), (C), and (D), the superimposed scaled current traces are for the S345C trimers C-S-S, C-C-S, and C-C-C. (E) Superimposed scaled current traces for double mutant S345C/H33Y. Control recordings were made for all mutants to monitor their degrees of densensitization (30 μM ATP was applied for 20-30 s). (F) Summary of percentage of block current in (A), (B), (C), (D) and (E) after applying 20 μM CdCl_2_. ** (*P*< 0.01), values are significantly different from those observed for rP2X2R-T and trimer C-S-S. * (*P*< 0.05), values are significantly different from those observed for rP2X2R-T and trimer C-S-S.

Our data for the double mutant H33C/S345C suggests that His33 and Ser345 are in close proximity for structural interaction when the channel is in the closed state. We questioned whether they were also within a few angstroms in the open state. One way to investigate this is to see whether the metal ion Cd^2+^ can be successfully coordinated between the cysteine side chains introduced at positions H33 and S345. Two previous studies have already investigated the effects of Cd^2+^ on the S345C mutant of P2X2R to coordinate Cd^2+^, but yielded contradictory results. One group observed no effect of Cd^2+^ on the ATP-gated current evoked through this mutant block [41]. Another group observed current block of S345C by Cd^2+^, but through the use of concatameric mutant receptors showed that this block was likely due to coordination of Cd^2+^ between the histidine at H33 and the substituted cysteine at S345C [35]. Histidine is thought commonly contribute to metal bridges with cysteine [42]. We sought to confirm whether His33 could coordinate Cd^2+^ with S345C, because if this was true it would suggest that these two side chains remain in close proximity in both the closed and open states. The rP2X2R-T (percentage of block current: 1.9% ± 0.3) and single mutant concatamer, Ser345 (C-S-S) (percentage of block current: 2.0% ± 0.4) were not inhibited by 20 μM Cd^2+^ (Fig. 6A and B). We also found that Cd^2+^ concentrations up to 2 mM did not inhibit the current amplitude of concatamer (S-S-S) and single mutant concatamer (C-S-S) (Fig. S4). However, the current amplitude of the two substituted cysteine concatamer (C-C-S) was also almost completely inhibited by Cd^2+^ (percentage of block current: 74.7% ± 3.6) (Fig. 6C). But surprisingly this effect was reversible. The current amplitude of three substituted cysteine concatamer (C-C-C) can be completely inhibited by Cd^2+^ (percentage of block current: 98.5% ± 1.5) (Fig. 6D). These data suggest that a less stable coordination formed in the two substituted cysteine concatamer than that in the three substituted concatamer. To test whether histidine was involved in the stable coordination of Cd^2+^ by mutants containing three S345C mutations we further mutated histidine to tyrosine at position 33. The current amplitude of the resulting double mutant, S345C/H33Y, was not inhibited by Cd^2+^ (percentage of block current: 15.2% ± 2.6) (Fig. 6E and F). This strongly suggests that His33 and S345 are close enough for the formation of a Cd^2+^ metal bridge. This means that from closed to open state the distance between His33 and Ser345 likely does not change substantially, which might explain why the current fold change of H33C/S345C before and after DTT incubation is small compare to V48C/I328C.

## Discussion

### Intra-subunit Interaction between His33 and Ser345

**Figure 7:**
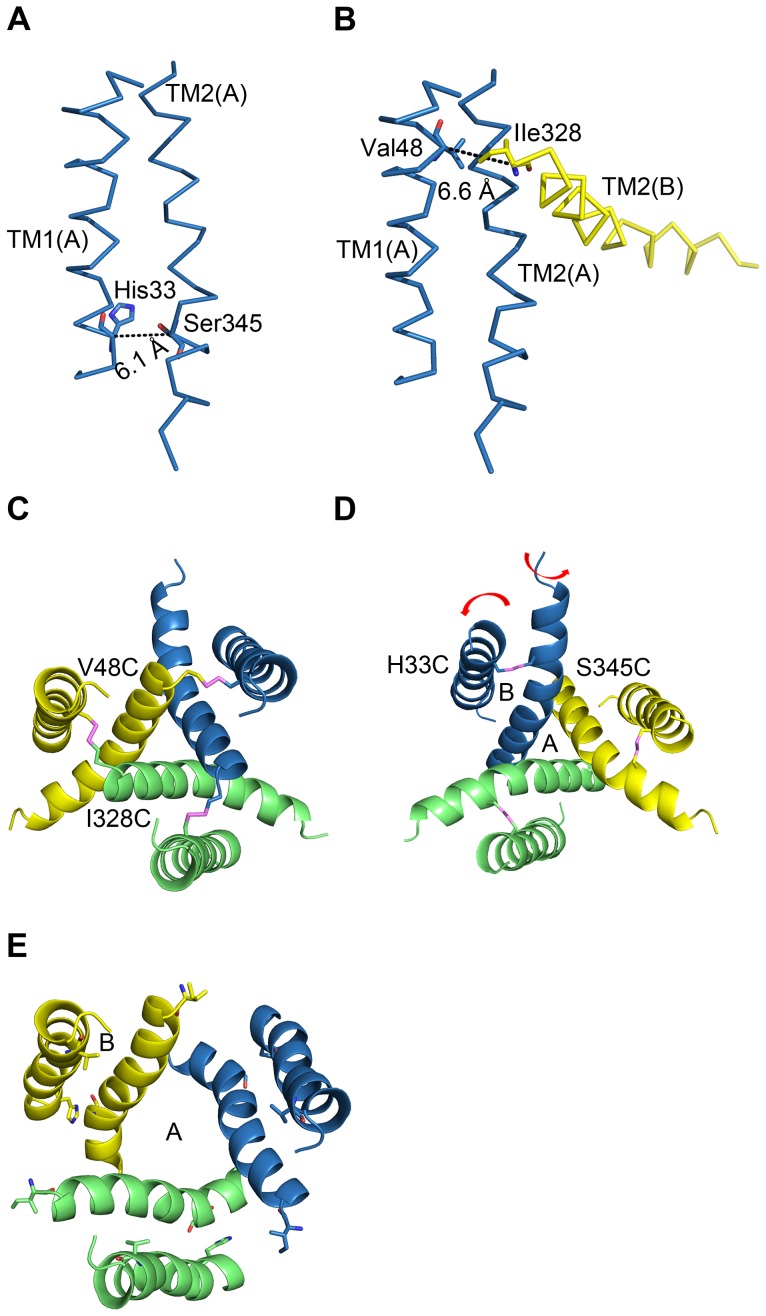
Homology models of the closed and open state of the rP2X2 receptor. (A) His33 and Ser345, which are involved in intra-subunit interactions in the closed state of rP2X2R, are shown in stick representation. The black dashed line shows the distance (6.1 Å) between the Cα atoms of His33 and Ser345. (B) Val48 and Ile328, which are involved in inter-subunit interactions in the closed state of rP2X2R, are shown in stick representation. The black dashed line shows the distance (6.6 Å) between the Cα atoms of Val48 and Ile328. For clarity, only subunit A and TM2 of subunit B are shown. The structure is viewed parallel to the membrane. (C) Inter-subunit disulfide bond formation between V48C and I328C in the closed state of rP2X2R. (D) Intra-subunit disulfide bond formation between H33C and S345C in the closed state of rP2X2R. The red arrow indicates that when ATP binds to the receptor, TM1 and TM2 rotate anticlockwise to open the pore. The trimer structure is viewed from the intracellular side (C) and the extracellular side (D). (E) The open state of rP2X2R. In (C), (D) and (E), the TMs of each subunit are in skyblue, lime, or yellow. The disulfide bridges are shown as violet sticks.

The central region of TM1 is close to the point of interaction between the two crossing TM helices [19]. After examining 36 pairs of double mutations, we found that reduction with DTT potentiated ATP-evoked currents in H33C/S345C, and that subsequent oxidation with H_2_O_2_ returned currents to their control amplitude (Fig. 1B and 1D). Four lines of evidence indicate an intra-subunit interaction between His33 and Ser345. First, after exposure to the reducing agent DTT, currents from the double mutant H33C/S345C were greatly enhanced (2 to 3 fold), indicating the formation of a disulfide bond when cysteines were present at both positions 33 and 345. However, previously enhanced current by DTT application could be reduced back to its initial amplitude by oxidation with H_2_O_2_, indicating that these residues are within 8.6 Å of each other in functioning receptors on the cell surface. This distance correlates well with the homology model of rP2X2R (which was built based on the recent crystal structure of zfP2X4.1R in the closed state). The homology model of rP2X2R revealed an average distance of ∼6.1 Å between the α-carbons of His33 and Ser345 (Fig. 7A). The second piece of evidence is that, for HEK293 cells expressing wild-type, the single mutants H33C and S345C, or the double mutants H33C/S345C, the detected proteins appeared as monomers under reducing and nonreducing conditions, consistent with results obtained for the single mutants V48C and I328C. In contrast, proteins obtained from HEK293 cells expressing V48C/I328C had prominent trimer bands when run under nonreducing conditions, but not when run under reducing conditions. As a positive control, we recapitulated previous functional studies showing that an inter-subunit disulfide bond forms between V48C and I328C. The distance between the side chains of Val48 and Ile328 was predicted to be ∼6.6 Å in our homology model of the closed state of the rP2X2 receptor (Fig. 7B), in line with that previously reported. The western blot results constitute a direct demonstration that H33C and S345C form an intra-subunit disulfide bond. The third piece of evidence is that the trimeric concatamer receptor, HC-CS-HS, in which only a single inter-subunit disulfide bond could possibly be formed, did not show any change in current amplitude after DTT incubation. In contrast, the concatamer mutants, CC-HS-HS and HC-CC-CS, in which only a single intra-subunit disulfide could possibly be formed, both demonstrated current potentiations in response to DTT exposure. However, both these single intra-subunit disulfide bonded concatamers showed much lower current increases in response to DTT than the concatamer containing three potential intra-subunit disulfide bonds (CC-CC-CC). These data support the inference that H33C and S345C form an intra-subunit disulfide bond and provide evidence that more disulfide bond formation sites in the intra-subunit (of the trimer concatamer) result in greater current potentiation after DTT incubation. This result also indicates that channel opening is partially inhibited by disulfide bond formation between His33 and Ser345. The fourth and final piece of evidence is that double mutant cycle analysis quantified the energy of the interactions between His33 and Ser345 on the basis of free energy changes (ΔΔG). These data suggest that the two residues can interact co-operatively within less than 7 Å [32]. In summary, multiple lines of evidence support the conclusion that His33 and Ser345 are in close proximity within the closed state of transmembrane domain of rP2X2R.

We observed that V48C/I328C currents increased 4 to 7-fold after DTT incubation, while the observed changes were only 2 to 3-fold for H33C/S345C. For both double mutants, the differences in EC_50_ values determined before and after DTT application may suggest that before DTT incubation the disulfide bond hinders the open-closed state (Fig. 7C and D). DTT incubation and breakage of the bond then allows the channel to open, normally. The DTT-induced change in the EC_50_ value determined for H33C/S345C (∼2-fold) is rather modest compared to the EC_50_ changes recorded for the V48C/I328C mutant (∼4-fold). This result might suggest that inter-subunit contacts are more critical than intra-subunit contacts in transmitting the binding site’s opening force to the transmembrane helices, but further investigation is required to confirm this hypothesis. According to the crystal structure of ATP-bound zfP2X4R [19], ATP binding may induce separation of adjacent subunits (Fig. 7E), which would increase the distance between V48C and I328C and explain why a disulfide bond between these positions would strongly hinder channel opening (Fig. 7B and C).

The close proximity of His33 and Ser345 and data from previous single mutant studies of these two residues in rat P2X2 receptor [43]-[44], led us to consider whether these two residues are also within close proximity in this narrow region of the open channel state. Our experiments concur with a previous study in showing that His33 is sufficiently close to position Ser345 in the open state as to contribute to form a stable Cd^2+^ bridge when cysteines are introduced into the latter positions in the receptor protein. The bond length of each S-Cd^2+^ is ∼2.5Å [42], [45], and so the distance between intra-subunit His33 and Ser345 positions in the open channel state is likely to be around ∼4.5 Å.

### Effects of Cysteine Pairs in the Transmembrane Domain

For the closed state of rP2X2R, no inter-subunit contacts between the TM1 and TM2 helices have been reported. In rP2X2R, 51 pairs of cysteines (including 15 pairs tested by Spelta et al.[20], [21]) cannot form disulfide bonds (Table 2). This may be due to numerous factors. To form a disulfide bond, two cysteines have to meet certain geometric constrains, such as distance and side-chain orientation. In proteins, the geometric requirements for disulfide bond formation imply that the distance between the respective α-carbons can be in the range of ∼4-7 Å. In addition, some dynamic factor, such as thermal mobility, may also affect disulfide bond formation. Hattori et al. [19] suggested that, based on the crystal structure of zfP2X4.1R, some inter-subunit contacts may exist between the TM1 and TM2 helices. However, we did not identify any inter-subunit contacts within TMDs in rP2X2R, which may suggest that interactions differ between different species and subtypes of P2XR. Hattori et al. concluded that the residues in the TM1 and TM2 helices are involved in intra-subunit interactions. Likewise, we identified an intra-subunit interaction between His33 and Ser345. It has been reported previously that the Gly29-Val61 and Asp338-Leu358 (rP2X4R numbering) regions are important in regulating the rate of channel deactivation by ivermectin [46], [47]. These results may further suggest that, in P2X2R or other subtypes, after the transition to the open state, the gaps between TM1 and TM2 likely constitute a site for interaction with lipids or allosteric modulators like ivermectin.

In summary, this work has, for the first time, identified intra-subunit interactions in transmembrane domains using substituted cysteine mutagenesis disulfide mapping and electrophysiological experiments and illustrates how the inter- and intra-subunit interactions affect channel opening.

## Supporting Information

Figure S1 **Transmembrane domains in P2X receptors.** (A) Schematic representation of the general features of P2X receptor subunits. Cys348, which is the only endogenous cysteine residue in the pore segment of TM2, was mutated to threonine, as indicated by a red circle. (B) Amino acid sequences of two transmembrane segments of rP2X2R, rP2X2R-T and zfP2X4R. Identical residues are shown in red. Cys348 was mutated to threonine, as indicated in yellow (rP2X2R-T).
(TIF)

Figure S2 **Initial study of rP2X2R and rP2X2R-T.** (A) Subcellular distribution of rP2X2R and rP2X2R-T 24 h after transfection. Scale bar is 10 µm. (B) Concentration effect of ATP on the 10-90% activation time for rP2X2R (•) and rP2X2R-T (○). (C) Relationship between 90-10% deactivation time and ATP concentration for rP2X2R (•) and rP2X2R-T (○), respectively, measured at all ATP concentrations. The dotted line indicates the mean value of rP2X2R-T responses at all ATP concentrations in (B) and (C). (D) ATP-evoked currents in HEK293 cells expressing rP2X2R-T. Each concentration of ATP (indicated below each current) was applied twice for 2s with similar results. The interval between each current was 3 min. (E) Concentration-response curve for rP2X2R (•) and rP2X2R-T (○). 30 μM ATP was applied before each test concentration to evaluate rundown. Data are shown as the mean peak current amplitude for each concentration of ATP divided by the mean amplitude of the peak response to the highest concentration of ATP (I/I_max_). The dotted line indicates that the value of I/I_max_ is equal to 0.5. Data points and error bars in this and all other figures represent the mean ± S.E.M. For detailed information on the EC_50_ in this and all other figures, see Table 3.
(TIF)

Figure S3 **Disulfide formation between TMDs.** (A) Effect of DTT and H_2_O_2_ on the V36C/S345C double mutant. After stable responses were evoked by 30 μM ATP (black bar), the cells were incubated in 10 mM DTT for 5 min (first arrow) and were then evoked by 30 μM ATP plus 10 mM DTT (white bar). After stable currents were obtained, cells were incubated with 0.3% H_2_O_2_ (second arrow) for 3 min to reverse the effects of DTT, after which the cells were evoked by 30 μM ATP plus 0.3% H_2_O_2_ (grey bar). The gaps indicate 3-min time intervals between ATP applications. For (B), (C), (D), (E), and (F), the same protocol was applied to the G30C/S345C, Q37C/S345C, H33C/G342C, H33C/C348, and H33C/I341C, respectively.
(TIF)

Figure S4 **Cd concentration-response relationship in two mutants.** (A) Superimposed scaled current traces show that rP2X2R-WT currents are not inhibited by applying 1 mM CdCl_2_. The control current trace (black) is evoked only by 30 μM ATP. For the test current trace (blue), 30 μM ATP was applied for 5s, after which the solution was switched to one containing 30 μM ATP plus 1 mM Cd^2+^ for 10–20s. Following this, we returned the cell to a solution containing only 30 μM ATP for 5s. The same protocol was applied to the other constructs in (B), (C), (D), and (E). In (B) and (C), 1 mM and 2 mM CdCl_2_ were applied to the trimer S-S-S, respectively. In (D) and (E), 1 mM and 2 mM CdCl_2_ were applied to the trimer C-S-S, respectively. Control recordings were made for all mutants to monitor their degrees of desensitization (30 μM ATP was applied for 20–30s).
(TIF)
