# Supplementary figures and images for: Functional Identification of Close Proximity Amino Acid Side Chains within the Transmembrane-Spanning Helixes of the P2X2 Receptor
Source: PLoS One. 2013 Aug 6;8(8):e70629. doi: 10.1371/journal.pone.0070629 (PMC3735612; doi:10.1371/journal.pone.0070629)

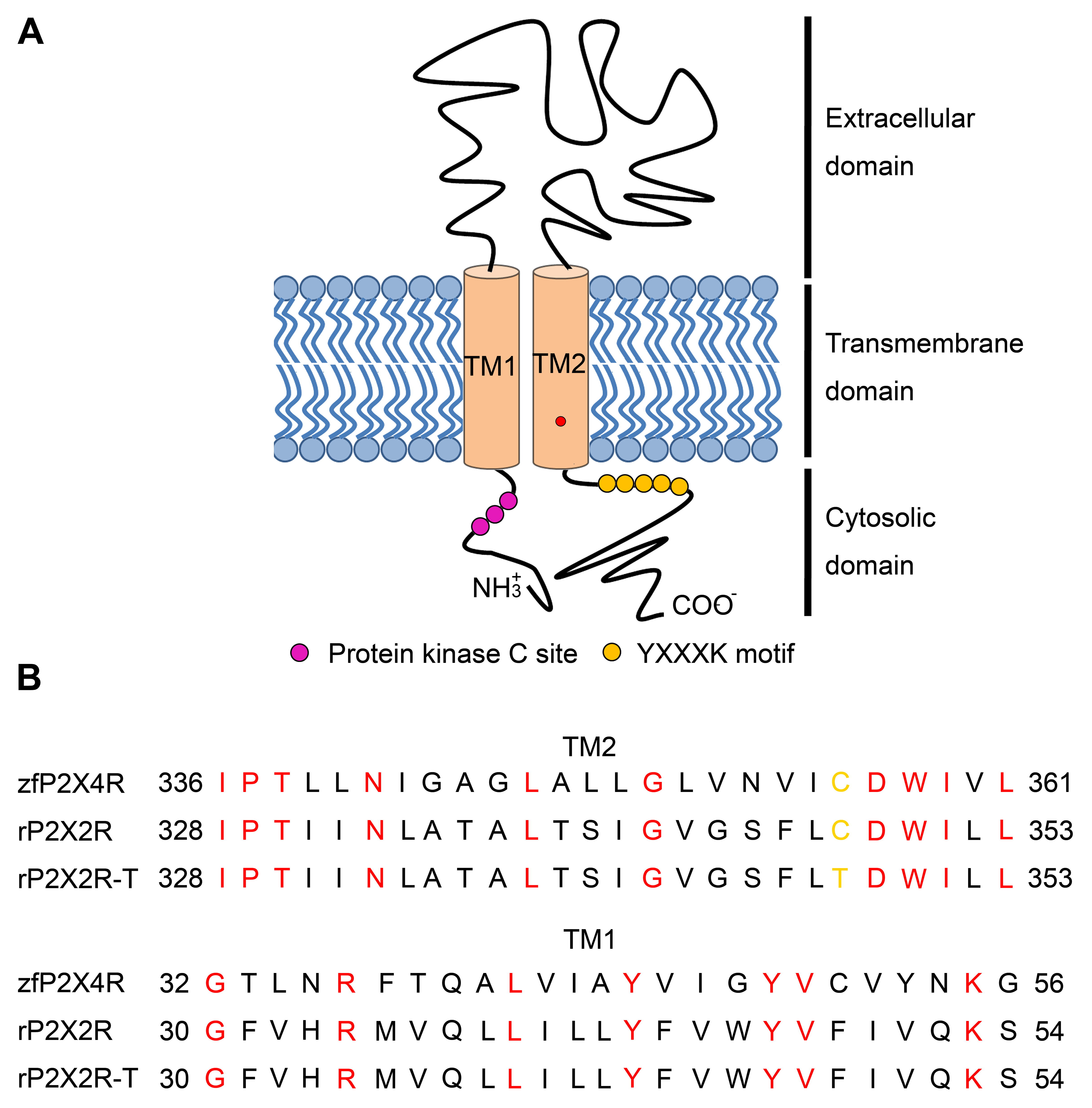

Supplement: Figure S1 — Transmembrane domains in P2X receptors. (A) Schematic representation of the general features of P2X receptor subunits. Cys348, which is the only endogenous cysteine residue in the pore segment of TM2, was mutated to threonine, as indicated by a red circle. (B) Amino acid sequences of two transmembrane segments of rP2X2R, rP2X2R-T and zfP2X4R. Identical residues are shown in red. Cys348 was mutated to threonine, as indicated in yellow (rP2X2R-T). (TIF) [file pone.0070629.s001.tif]

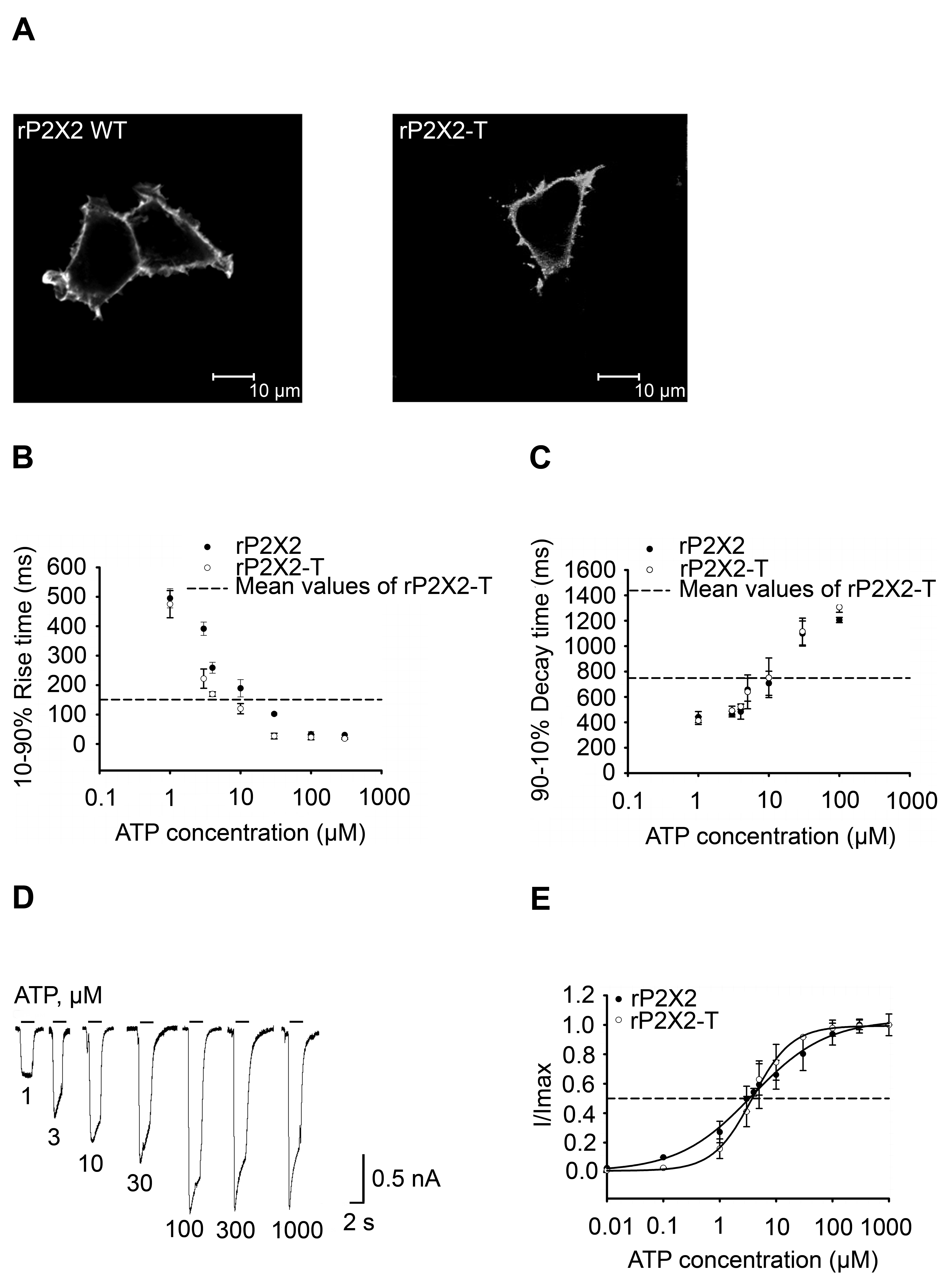

Supplement: Figure S2 — Initial study of rP2X2R and rP2X2R-T. (A) Subcellular distribution of rP2X2R and rP2X2R-T 24 h after transfection. Scale bar is 10 µm. (B) Concentration effect of ATP on the 10-90% activation time for rP2X2R (•) and rP2X2R-T (○). (C) Relationship between 90-10% deactivation time and ATP concentration for rP2X2R (•) and rP2X2R-T (○), respectively, measured at all ATP concentrations. The dotted line indicates the mean value of rP2X2R-T responses at all ATP concentrations in (B) and (C). (D) ATP-evoked currents in HEK293 cells expressing rP2X2R-T. Each concentration of ATP (indicated below each current) was applied twice for 2s with similar results. The interval between each current was 3 min. (E) Concentration-response curve for rP2X2R (•) and rP2X2R-T (○). 30 μM ATP was applied before each test concentration to evaluate rundown. Data are shown as the mean peak current amplitude for each concentration of ATP divided by the mean amplitude of the peak response to the highest concentration of ATP (I/Imax). The dotted line indicates that the value of I/Imax is equal to 0.5. Data points and error bars in this and all other figures represent the mean ± S.E.M. For detailed information on the EC50 in this and all other figures, see Table 3. (TIF) [file pone.0070629.s002.tif]

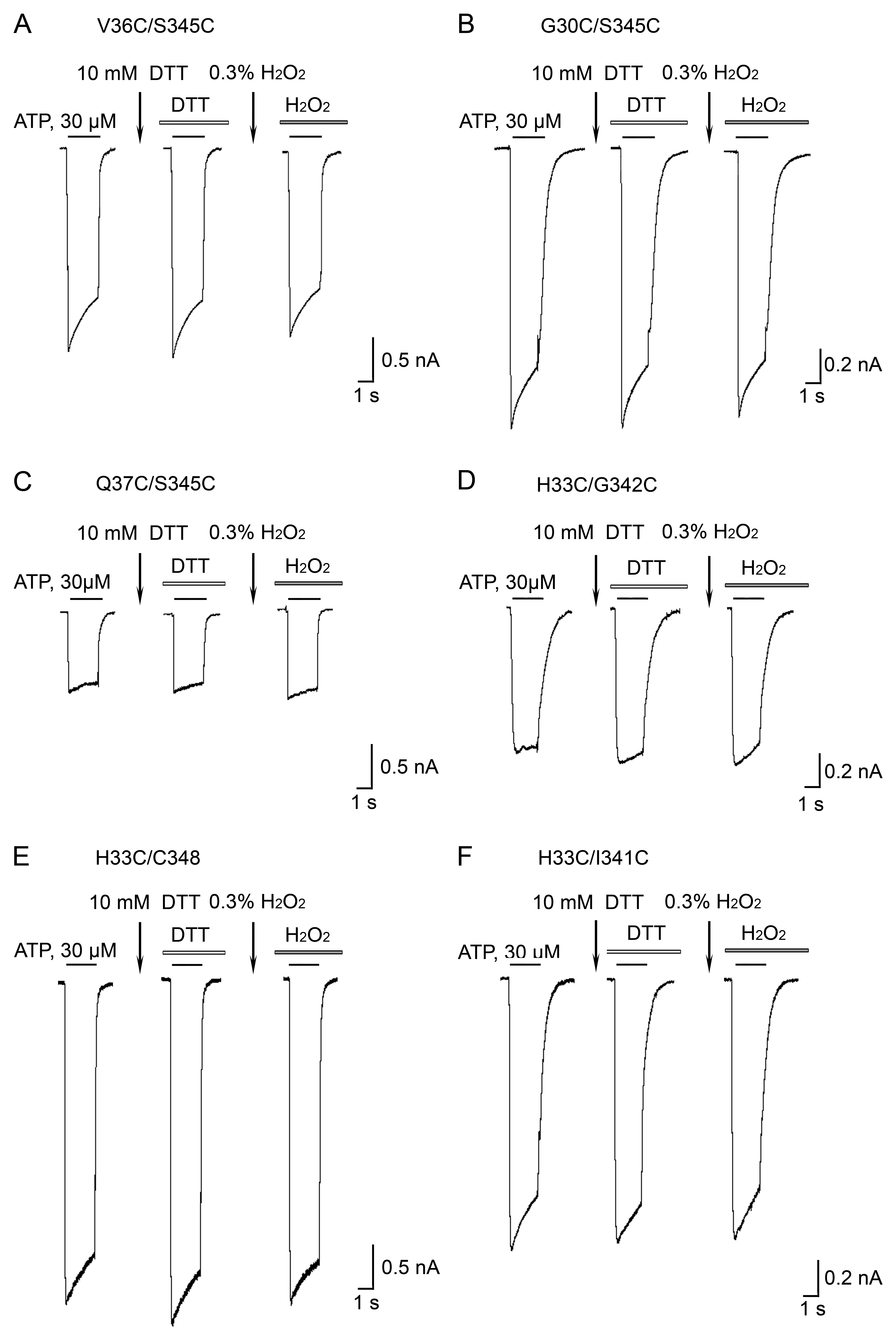

Supplement: Figure S3 — Disulfide formation between TMDs. (A) Effect of DTT and H2O2 on the V36C/S345C double mutant. After stable responses were evoked by 30 μM ATP (black bar), the cells were incubated in 10 mM DTT for 5 min (first arrow) and were then evoked by 30 μM ATP plus 10 mM DTT (white bar). After stable currents were obtained, cells were incubated with 0.3% H2O2 (second arrow) for 3 min to reverse the effects of DTT, after which the cells were evoked by 30 μM ATP plus 0.3% H2O2 (grey bar). The gaps indicate 3-min time intervals between ATP applications. For (B), (C), (D), (E), and (F), the same protocol was applied to the G30C/S345C, Q37C/S345C, H33C/G342C, H33C/C348, and H33C/I341C, respectively. (TIF) [file pone.0070629.s003.tif]

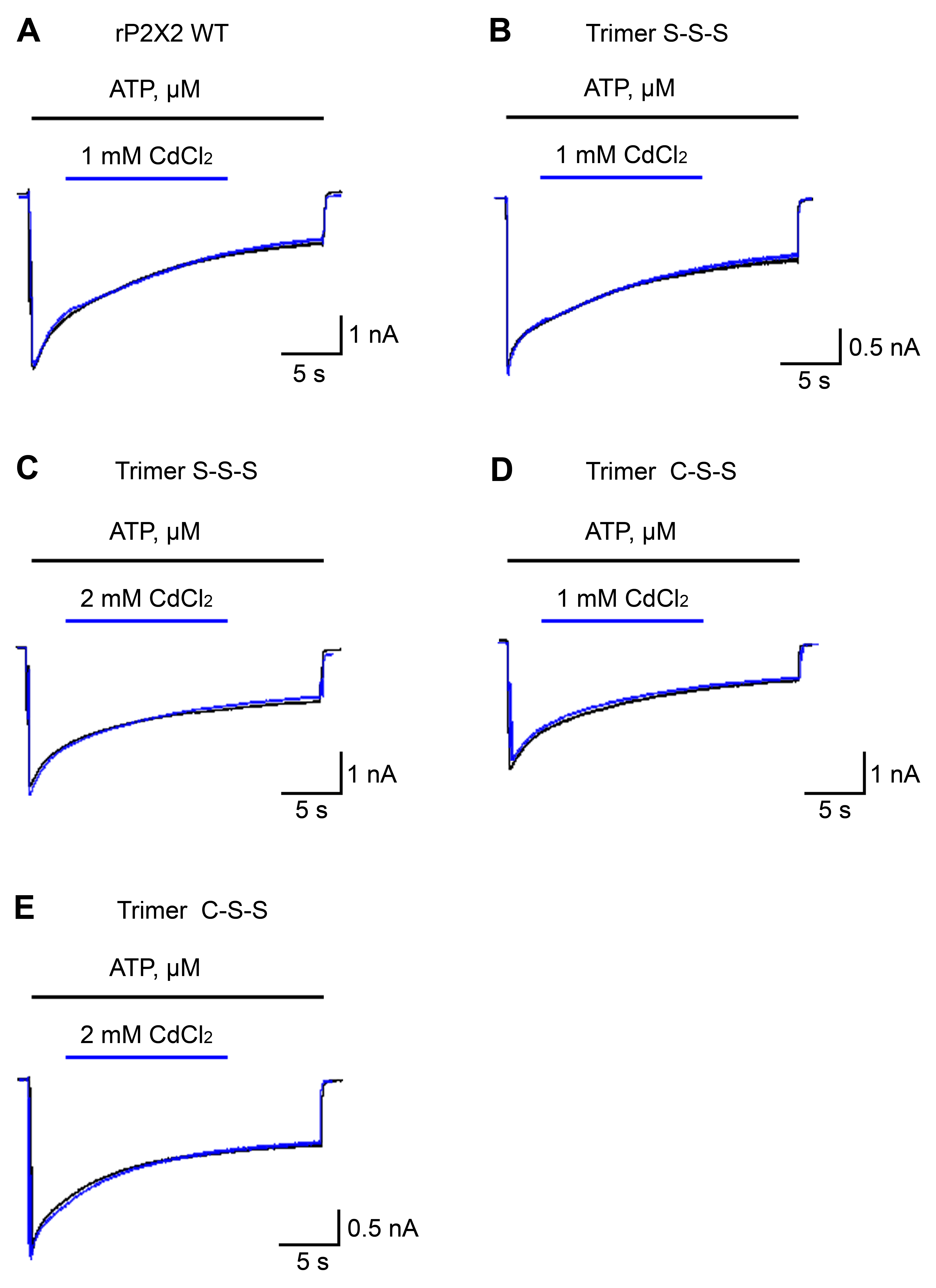

Supplement: Figure S4 — Cd concentration-response relationship in two mutants. (A) Superimposed scaled current traces show that rP2X2R-WT currents are not inhibited by applying 1 mM CdCl2. The control current trace (black) is evoked only by 30 μM ATP. For the test current trace (blue), 30 μM ATP was applied for 5s, after which the solution was switched to one containing 30 μM ATP plus 1 mM Cd2+ for 10–20s. Following this, we returned the cell to a solution containing only 30 μM ATP for 5s. The same protocol was applied to the other constructs in (B), (C), (D), and (E). In (B) and (C), 1 mM and 2 mM CdCl2 were applied to the trimer S-S-S, respectively. In (D) and (E), 1 mM and 2 mM CdCl2 were applied to the trimer C-S-S, respectively. Control recordings were made for all mutants to monitor their degrees of desensitization (30 μM ATP was applied for 20–30s). (TIF) [file pone.0070629.s004.tif]
